# Low Urinary Iodine Concentrations Associated with Dyslipidemia in US Adults

**DOI:** 10.3390/nu8030171

**Published:** 2016-03-17

**Authors:** Kyung Won Lee, Dayeon Shin, Won O. Song

**Affiliations:** Department of Food Science and Human Nutrition, Michigan State University, 469 Wilson Road, Trout FSHN Building, East Lansing, MI 48824, USA; kyungwon@msu.edu (K.W.L.); shinda@msu.edu (D.S.)

**Keywords:** iodine, serum lipids, cholesterol, dyslipidemia, NHANES

## Abstract

Iodine is an essential component of the thyroid hormone which plays crucial roles in healthy thyroid function and lipid metabolism. However, the association between iodine status and dyslipidemia has not been well established at a population level. We aimed to test the hypothesis that the odds of dyslipidemia including elevated total cholesterol, triglycerides, low-density lipoprotein (LDL) cholesterol and apolipoprotein B, and lowered high-density lipoprotein (HDL) cholesterol and HDL/LDL ratio are associated with urinary iodine concentration (UIC) in a population perspective. Data of 2495 US adults (≥20 years) in the National Health and Nutrition Examination Survey 2007–2012 were used in this study. Two subgroups (*i.e.*, UIC below *vs.* above the 10th percentile) were compared of dyslipidemia as defined based on NCEP ATP III guidelines. The differences between the groups were tested statistically by chi-square test, simple linear regressions, and multiple logistic regressions. Serum lipid concentrations differed significantly between two iodine status groups when sociodemographic and lifestyle covariates were controlled (all, *p* < 0.05). Those with the lowest decile of UIC were more likely to be at risk for elevated total cholesterol (>200 mg/dL) (adjusted odds ratio (AOR) = 1.51, 95% confidence interval (CI): 1.03–2.23) and elevated LDL cholesterol (>130 mg/dL) (AOR = 1.58, 95% CI: 1.11–2.23) and lowered HDL/LDL ratio (<0.4) (AOR = 1.66, 95% CI: 1.18–2.33), compared to those with UIC above the 10th percentile. In US adults, low UIC was associated with increased odds for dyslipidemia. Findings of the present cross-sectional study with spot urine samples highlight the significant association between UIC and serum lipids at population level, but do not substantiate a causal relationship. Further investigations are warranted to elucidate the causal relationship among iodine intakes, iodine status, and serum lipid profiles.

## 1. Introduction

Iodine is an indispensable component of thyroid hormone biosynthesis and normal thyroid function [1]. Thyroid hormone plays a key role in the regulation of multiple mechanisms, particularly lipid synthesis and absorption [2]. Since every cell in the body is affected by thyroid hormone, iodine status is associated with various health outcomes including goiter, hypothyroidism, mental retardation, and dyslipidemia [3]. Impaired lipid metabolism may be resulted from inadequate thyroid hormone production due to insufficient iodine [4]. In an iodine deficient population, serum thyroid stimulating hormone (TSH) levels is elevated to prompt an uptake of circulating iodine by the thyroid [5]. It has been reported that elevated TSH has a positive association with risk for dyslipidemia [6]. Additionally, abnormal thyroid hormone due to iodine deficiency may lead to adverse aftereffects such as hypothyroidism throughout various life stages [7]. It is well-known that hypothyroidism is a major risk factor for the development of dyslipidemia, hypercholesterolemia and cardiovascular disease (CVD) [8,9]. Hypothyroidism accelerates elevations in serum total cholesterol and low-density lipoprotein (LDL) cholesterol by increasing cholesterol absorption in the intestines and lowering LDL cholesterol clearance from the serum. Moreover, hypothyroidism may increase serum triglycerides by decreasing lipoprotein lipase activity [10,11].

Dyslipidemia broadly describes abnormalities associated with serum lipid metabolism. Its clinical consequences are elevated levels of total cholesterol, triglycerides, and LDL cholesterol, and decreased high-density lipoprotein (HDL) cholesterol levels [12]. Dyslipidemia is one of the risk factors of CVD and a main component of metabolic syndrome. Increased social burdens resulting from CVD and metabolic syndrome have led to extensive investigations to prevent them in the field of nutrition [13,14]. Dyslipidemia consisting of elevated total cholesterol and LDL cholesterol has been observed in patients with hypothyroidism [15,16]. The majority of previous studies noted that abnormalities in total cholesterol and LDL cholesterol were normalized after the successful treatment of hypothyroidism [17,18]. Although many studies examined the association between thyroid dysfunction and lipid levels, we still have no adequate information regarding the relationship between iodine status and serum lipid levels. Only a very few studies have investigated the development of dyslipidemia in relation to iodine status. Uncontrolled and small scale studies have been conducted on the effects of iodine treatments in goitrous subjects with CVD and lipid abnormalities. Previous studies reported that iodine treatments for subjects with goiter resulting from iodine deficiency improved serum lipid profiles by lowering previously elevated total cholesterol and LDL cholesterol [19,20]. In a recent study with mice, hypothyroidic function due to insufficient iodine intake changed lipid profiles, while excessive iodine intake had a beneficial effect on lipid metabolism [21].

Considering that the prevalence of both iodine deficiency and dyslipidemia still remain high among some subgroups in the US [22,23], further research is appropriate to elucidate the association between iodine status and lipids. It takes a long time to develop dyslipidemia from hypothyroidism [11,24] and hypothyroidism from iodine deficiency [25]. If dyslipidemia is associated with inadequate iodine status, the correction for iodine status may have considerable benefits for prevention and treatment of dyslipidemia. Therefore, we hypothesized that urinary iodine concentration (UIC) is associated with the odds of dyslipidemia in the US population. The specific aims of our study were to identify sociodemographic and lifestyle variables affecting UIC and serum lipids, to determine the association of UIC with serum lipid levels, and to estimate risks for dyslipidemia by UIC using recently available National Health and Nutrition Examination Survey (NHANES) data collected from 2007 to 2012. UIC from a spot urine sample is the only indicator of iodine status that is currently available in the NHANES. UIC is a reliable indicator for the iodine status assessment, because more than 90% of dietary iodine appears in urine [26]. UIC from ten repeat collections from spot urine samples or 24-h urine samples can be applied to as a measurement for individual’s iodine status, whereas single spot urine samples should be used to assess iodine status only at a population level [22,27]. Although it is hard to conclude anything at the individual level due to limitation of indicators for iodine status in the NHANES, we can describe an association between below *vs.* above the 10th percentile of UIC subgroups with serum lipid concentrations based on large population-based analyses.

## 2. Methods

### 2.1. Data Source and Study Sample

The NHANES, a cross-sectional examination survey, is conducted by the National Center for Health Statistics (NCHS) of the Centers for Disease Control and Prevention (CDC). Each NHANES survey is based on a complex, stratified, multistage, and probability cluster designed to obtain nationally representative samples of civilian, noninstitutionalized residents in the US [28]. The NHANES consists of interviews, laboratory tests, and physical examinations administered by highly trained staff [29]. NHANES protocols were approved by the NCHS Research Ethics Review Board and all subjects aged ≥18 years participated voluntarily after giving their informed consent [30]. Detailed descriptions of survey plan and design have been previously provided in the NHANES analytic guidelines [28] and thus will not be explained here.

In 1999, the NHANES became a continuous survey with collecting data in every two-year cycles. In this study, we merged three continuous NHANES cycles (2007–2008, 2009–2010, and 2011–2012) and focused on 9164 US adults (≥20 years) who participated in the NHANES 2007–2012 and have urinary iodine information. We excluded those who were pregnant and lactating women (*n* = 267), not eligible for data analyses because of missing information on serum lipids (*n* = 4871), those who were taking any medications for thyroid dysfunction or dyslipidemia (*n* = 824), and those with missing information on key sociodemographic and lifestyle characteristics (*n* = 707). The final analytic sample consisted of 2495 adults.

### 2.2. Definition of Dyslipidemia

We utilized NHANES serum laboratory information on total cholesterol, triglycerides, HDL cholesterol, LDL cholesterol, and apolipoprotein B levels to assess dyslipidemia. For this study, dyslipidemia was defined based on the guidelines provided in the third report of the US National Cholesterol Education Programme Adult Treatment Panel III (NCEP ATP III) [31]: total cholesterol >200 mg/dL for elevated total cholesterol; triglycerides >150 mg/dL for elevated triglycerides; HDL cholesterol <40 mg/dL in men and <50 mg/dL in women for lowered HDL cholesterol; LDL cholesterol >130 mg/dL for elevated LDL cholesterol; HDL/LDL ratio <0.4 for lowered HDL/LDL ratio; apolipoprotein B >130 mg/dL for elevated apolipoprotein B.

### 2.3. Iodine Status

The NHANES has included measured UIC to monitor the iodine status among US population aged 6 years and older since 1971. Information on UIC has been collected through spot urine samples by the Elemental Analysis Laboratory of the CDC’s Division of Laboratory Sciences. UIC was measured by inductively coupled plasma mass spectroscopy (ICP-MS) based on the method by Caldwell *et al.* [32,33] using the same equipment at the same laboratories [34,35]. To examine the association of UIC and serum lipid levels, the participants included in this study were divided into deciles according to UIC distribution and the lowest decile of them was defined as having low UIC (9.0–47.2 μg/L). Then, serum lipid levels were compared between two subgroups (below *vs.* above the 10th percentile of UIC) [36].

### 2.4. Covariates

The NHANES included information collected on the sociodemographic and lifestyle characteristics through interviews administered by trained interviewers. In this study, variables included in the statistical analytic models were sex (men and women); age (20–39, 40–59, and ≥60 years); race/ethnicity (non-Hispanic whites, non-Hispanic blacks, all Hispanics, and others); education (less than high school, high school, and more than high school); family poverty income ratio (PIR) (low, ≤1.85; medium, 1.85 to ≤3.5; and high >3.5) [28]; supplement use (reported use of a dietary supplement in the last 30 days; yes and no) [37]; smoking (serum cotinine level; low, <0.015 mg/L; medium, 0.015 to <10 mg/L; high, ≥10 mg/L) [38]; alcohol consumption (average number of drinks/day; no drink, >0 to <1, 1 to <2, and ≥2 drinks/day) [37]; BMI (underweight, <18.5 kg/m^2^; normal, 18.5 to <25 kg/m^2^; overweight, 25 to <30 kg/m^2^; and obese, ≥30 kg/m^2^) [39]; physical activity (Metabolic Equivalent Task (MET)-minutes/week) from leisure-time physical activity: no activity, 0 to <500, 500 to <1000, and ≥1000 MET-minutes/week [40].

### 2.5. Statistical Analyses

We analyzed all data with SAS (version 9.3, SAS Institute Inc, Cary, NC, USA). To account for complex survey design, survey nonresponse, and planned oversampling, we used SURVEY procedure including sample weight, stratum (SDMVSTRA), and primary sampling unit (SDMVPSU) recommended by NCHS for the NHANES analysis [29].

The chi-square test was performed to investigate the distributions (%) and the association between UIC and sociodemographic and lifestyle variables (categorical variables) (Table S1). We used bivariate methods based on simple linear regressions to test the differences in serum lipid profiles by sociodemographic and lifestyle variables (Table 1 and Table 2). The variables that were significantly associated were considered as covariates in subsequent analyses. Serum lipid levels including total cholesterol, triglycerides, HDL cholesterol, LDL cholesterol, HDL/LDL ratio and apolipoprotein B (continuous variables) were compared between two subgroups (*i.e.*, below *vs.* above the 10th percentile of UIC) using linear regression (Table 3 and Table S2). Multiple logistic regression analyses were used to determine risks for abnormal lipid levels according to UIC (Table 4). Odds ratios (ORs) with 95% confidence intervals (CIs) were calculated after controlling for covariates in two models: unadjusted (model 1) and adjusted for sex, age, race/ethnicity, education, income, supplement use, smoking, alcohol consumption, BMI, and physical activity (model 2). Two-sided *p* values were considered to be statistically significant if *p* < 0.05.

## 3. Results

The sociodemographic and lifestyle characteristics of the study population categorized by UIC are presented in Table S1. Compared to those with UIC above the 10th percentile, those with UIC below the 10th percentile were more likely to be women, have a poorer level of education, and less likely to take iodine-containing supplements and to be obese.

Unadjusted biomarker concentrations of serum lipid according to sociodemographic and lifestyle characteristics are shown in Table 1 and Table 2. Among the sociodemographic variables, sex, age, race/ethnicity, and education (except for total cholesterol) were significantly associated with the majority of serum lipid biomarkers, except for LDL cholesterol which was associated only with age (Table 1). Unlike other sociodemographic variables, PIR was associated only with HDL cholesterol levels. Almost all of the lifestyle variables also had a significant relation with serum lipid status biomarkers (Table 2). Particularly, alcohol consumption and BMI were significantly associated with all five serum lipid biomarkers. The lifestyle variables, supplement use and smoking (except for LDL cholesterol and apolipoprotein B), and physical activity (except for total cholesterol and LDL cholesterol) were also significantly associated with most lipid biomarker concentrations. In our bivariate model, sex, age, and BMI explained the largest portion of the variability in the majority of serum lipid biomarkers; and other sociodemographic and lifestyle variables accounted for little variance in most serum lipid biomarkers.

Serum lipid levels by UIC are shown in Table 3 and Table S2. In men, serum lipid levels did not differ according to UIC, whereas in women, two serum lipid biomarkers differed significantly by UIC. Particularly in older women (≥60 years), serum lipid levels such as total cholesterol, LDL cholesterol, and apolipoprotein B were significantly different according to UIC (all, *p* < 0.05). Older women with low UIC had a higher level of total cholesterol, LDL cholesterol and apolipoprotein B levels (231.6 ± 7.3 mg/dL, 151.8 ± 5.6 mg/dL and 107.8 ± 5.6 mg/dL, respectively) than those with UIC above the 10th percentile (215.4 ± 4.3 mg/dL, 132.2 ± 3.2 mg/dL and 96.2 ± 2.9 mg/dL, respectively).

The adjusted ORs and 95% CIs for risk factors of dyslipidemia after adjustment for covariates by UIC are described in Table 4. In comparison to adults with UIC above the 10th percentile, adults with the lowest decile of UIC were more likely to have dyslipidemia. The adjusted ORs of dyslipidemia risk factors among adults with low UIC *vs.* UIC at or above the 10th percentile were 1.63 (95% CI, 1.16–2.28) for elevated LDL cholesterol (>130 mg/dL) and 1.71 (95% CI, 1.09–2.67) for lowered HDL/LDL ratio (<0.4). Adults with low UIC had increased risk of elevated total cholesterol (>200 mg/dL) at marginally significant levels (*p* = 0.0666).

## 4. Discussion

In this study, significant differences were found in multiple lipid measures according to UIC. Majority of adults with UIC above the 10th percentile had desirable serum lipid range, whereas, those with UIC below the 10th percentile had elevated total cholesterol, LDL cholesterol, and apolipoprotein B. Our findings are consistent with previous researches regarding lipid profiles related to iodine status and iodine treatments in European [19,41] and African countries [42]. The research on 136 adolescents with endemic goitrous in Germany demonstrated that those with iodine deficiency-induced goiter had abnormally higher average total cholesterol and LDL cholesterol levels compared to non-goitrous control subjects [19]. In another study conducted by the same research group, the effects of iodine treatment for six months improved serum lipid levels [41]. Iodine supplement treatment for euthyroid goiter on children resulted significant decrease in total cholesterol and LDL cholesterol to their normal level [43]. A recent randomized controlled intervention study also reported that 6-month intervention with iodine supplementation significantly decreased risks for hypercholesterolemia in overweight and obese Moroccan women [44]. Additionally, a rural African population-based study reported that pregnant women and their offspring in an iodine deficient area are more likely to have a risk of coronary complications and hyperlipidemia compared to non-iodine deficient region [42]. Empirical evidence from clinical studies has demonstrated that iodine treatment was successful for those with CVD and high blood pressure [45]. There have also been many studies on thyroid dysfunction as a risk factor of lipid abnormalities and CVD. In adolescents with type 1 diabetes, subclinical hypothyroidism was positively correlated with mild dyslipidemia and increased the risk of CVD [46]. In 40 Spanish children aged 2–9 years, mean HDL cholesterol level was significantly lower in children with hypothyroidism compared to healthy children [47]. As thyroid failure has become one of determinants for dyslipidemia, an assertion has been raised that biochemical screening for thyroid disorders is needed for every dyslipidemia patient [48]. These existing studies suggest that long-term inadequate iodine intake may increase risks for dyslipidemia and CVD even though no clinical sign of thyroid dysfunction are present. We significantly expanded the scope of prior study using data from the representative US population and found that this association is still significant among US adults.

No association was found between TSH and UIC except for the case of excessive iodine intake where TSH levels were elevated by inhibiting thyroid hormone synthesis, which is known as the Wolff-Chaikoff effect [32,49]. It is hard to find UIC associated with TSH, particularly in the case of low UIC. Low UIC stimulates thyroid auto-regulation and an offsetting increase in serum triiodothyronine (T3), which normalize thyroid function [50]. Therefore, thyroid function test measuring TSH and thyroxine (T4) levels cannot be a good indicator for reflecting iodine status. Even though insufficient iodine intakes assessed by a single spot urine sample is not necessarily consistent with chronic iodine deficiency or subclinical hypothyroidism, persistent iodine deficiency leads to decreased thyroid hormone synthesis and elevated TSH easily with low-normal serum T4 concentration and high-normal serum T3 range [6]. As mentioned earlier, thyroid hormone is closely link to many metabolic pathways. Thus, the decrease in thyroid hormone synthesis induced by inadequate iodine intake raises the possibility of abnormal lipid metabolism [4]. In addition to classic pathway of lipid metabolism related to thyroid hormone, thyroid hormone-independent effect of TSH on lipid abnormalities was observed [51,52]. Lowered activity of hepatic lipase, which plays an important role in modulation of lipid levels and promoting cholesterol uptake by the liver, due to elevated TSH levels directly affect serum lipid levels [53]. In accordance with this mechanism, biochemical responses iodine deficiency may produce a more atherogenic lipid and higher risk for CVD [54]. Although iodine is also an essential component of the thyroid hormone and healthy thyroid function, iodine itself may play an important role in the cardiovascular health such as cancer-fighting properties and anti-inflammation [55]. However, only limited data support the proposition that iodine status was inversely associated with risk for CVD and iodine intakes were the largest contributor to mortalities due to CVD [20]. The underlying mechanism influencing serum lipid abnormalities by iodine status still remains partially unclear. A perspective and population-based study is required to elucidate a mechanism connected to iodine deficiency, thyroid function, and serum lipid. Therefore, continual monitoring of the independent role of iodine status in dyslipidemia allows us to put a new perspective on the treatment and possible prevention of CVD as well as dyslipidemia.

This study has several limitations. An important limitation of the study is that our findings are based on the cross-sectionally designed survey data. Such a fact makes it difficult to determine a causal relationship between UIC and dyslipidemia. However, this study highlighted that those with low UIC had increased risk of lipid abnormalities. In an animal study conducted by Zhao *et al.* [21], they confirmed the decreasing effect of high iodine intake on hyperlipidemia in mice, but did not investigate that kind of effect in humans. Therefore, adding to our results, it will be necessary to make further efforts to identify a casual effect of high iodine exposure on lipid concentration among those with iodine excess. Investigating potential mechanism between a wide range of iodine intake and lipid levels can provide us with a better understanding of the role of iodine status in pathogenesis of lipid metabolism. Another limitation is the lack of key information on iodine intake. Spot urine sample UIC is the only indicator for iodine status measurement in the NHANES. It has been well-documented that UIC obtained from spot urine sample is a suitable indicator for assessment of population’s iodine status, but not for individual’s [27]. Moreover, assessing iodine status based on UIC could not determine the actual amount of iodine intake from dietary sources. To overcome recognized limitations, we used the decile approach. Using this approach, we could have significant findings regarding the association between UIC and serum lipids. However, findings from decile grouping of study samples’ UIC distribution still have the limitation that inconsistent results can be found with the different populations. Therefore, their association should be interpreted with circumspection. Understanding the impact of actual dietary iodine intakes and individual’s iodine status on serum lipids is worth further investigation as well. Lastly, the study using mice reported that the effect of iodine intake on lipid metabolism may vary depending on the sex of the mice, mentioning needs of considering interaction between iodine status and the sex hormone in the lipid metabolism [21]. Further investigation is needed to explore the effect of these interactions. Adding the strengths of the present study mentioned above, this is a recent and unique study investigating the association between UIC and serum lipid profiles based on larger samples of the US adult population. We confirmed that sociodemographic and lifestyle factors affect UIC and serum lipids, the significant association between low UIC and lipid profiles persisted even after controlling for those confounding factors in our analysis models.

## 5. Conclusions

In summary, according to the NHANES 2007–2012, we found a cross-sectional association between low UIC measured by a spot urine sample and serum lipid profiles, particularly among total cholesterol, LDL cholesterol, and apolipoprotein B. Thus, efforts for maintaining adequate level of urinary iodine are recommended to prevent risk factors for dyslipidemia among US adults. However, there is a limitation in that, even though UIC used in this study as a biomarker might capture iodine status at the population level, but not at the individual level. Therefore, there is a need for further study investigating the relationship between biomarkers for iodine status that can reflect the iodine status of individuals and serum lipid levels. Additionally, further prospective research regarding the effect of iodine status on lipid metabolism and vice versa will establish epidemiological evidence for a causal relationship between them and help develop effective educational and clinical interventions in this area.

## Figures and Tables

**Table 1:** Unadjusted serum lipid status biomarker levels by sociodemographic variable categories for US adults, NHANES 2007–2012 ^1^.

Sociodemographic Variable		TC	TG	HDL-C	LDL-C	Apo B
		mg/dL	mg/dL	mg/dL	mg/dL	mg/dL
Sex	Men	196.8 ± 1.3 ^2,^*	130.5 ± 2.3 **	48.9 ± 0.5 **	121.8 ± 1.2	95.1 ± 0.9 *
	Women	201.6 ± 1.5	112.0 ± 2.6	59.8 ± 0.7	119.4 ± 1.3	91.4 ± 1.0
	*r^2^*, *%* ^3^	<1	2.12	11.31	<1	<1
Age	20–39 years	184.8 ± 1.4 **	112.8 ± 2.8 **	51.6 ± 0.7 **	110.6 ± 1.3 **	85.9 ± 0.9 **
	40–59 years	208.3 ± 1.7	129.0 ± 2.1	54.7 ± 0.8	127.8 ± 1.6	98.7 ± 1.1
	≥60 years	212.6 ± 2.7	123.8 ± 3.1	59.9 ± 1.2	127.9 ± 2.3	98.3 ± 1.3
	*r*^2^, *%*	9.80	1.41	3.06	6.20	6.85
Race/Ethnicity	NHW	200.5 ± 1.3 *	122.0 ± 2.2 **	54.7 ± 0.7 **	121.4 ± 1.1	93.4 ± 0.9 *
	NHB	193.5 ± 2.1	97.1 ± 3.9	58.1 ± 1.1	116.0 ± 1.9	90.1 ± 1.7
	All Hispanics	197.1 ± 1.6	133.6 ± 2.4	49.8 ± 0.6	120.5 ± 1.3	95.6 ± 1.1
	Other	197.5 ± 4.1	128.2 ± 9.8	52.8 ± 1.5	119.1 ± 3.2	92.3 ± 3.0
	*r*^2^, *%*	<1	2.12	1.75	<1	<1
Education	Less than high school	199.0 ± 1.9	127.9 ± 2.5 **	50.5 ± 0.8 **	122.9 ± 1.5	96.7 ± 1.2 **
	High school	200.7 ± 2.1	129.5 ± 3.4	52.0 ± 1.0	122.8 ± 1.8	96.4 ± 1.4
	More than high school	198.6 ± 1.6	116.5 ± 2.3	56.1 ± 0.5	119.2 ± 1.3	91.2 ± 0.9
	*r*^2^, *%*	<1	<1	2.16	<1	1.20
PIR	Low	198.1 ± 1.6	124.9 ± 2.2	52.8 ± 0.7 *	120.3 ± 1.4	93.8 ± 0.9
	Medium	198.0 ± 2.3	118.1 ± 3.5	53.7 ± 1.0	120.7 ± 2.0	93.5 ± 1.6
	High	200.6 ± 1.7	120.5 ± 2.6	55.7 ± 0.8	120.9 ± 1.4	92.9 ± 0.9
	*r*^2^, *%*	<1	<1	<1	<1	<1

^1^ Data are from the National Health and Nutrition Examination Surveys. All data except for sample size are weighted accounting for the complex study design according to the directions of the National Center for Health Statistics. Biomarker levels represent mean ± SEM. The total *n* size was 2495. TC, total cholesterol; TG, triglyceride; HDL-C, HDL cholesterol; LDL-C, LDL cholesterol; Apo B, apolipoprotein B; NHW, non-Hispanic white; NHB, non-Hispanic black; PIR, family poverty-income ratio (low: 0–1.85; medium: 1.85 < to 3.5; high: >3.5); ^2^ *p* values were based on Wald F test, which tests whether at least 1 of the means across the sociodemographic variable categories is significantly different (* *p* < 0.05, ** *p* < 0.01); ^3^ values for *r*^2^ are based on model 1, simple linear regression, by categories as shown.

**Table 2:** Unadjusted serum lipid status biomarker levels by lifestyle variable categories for US adults, NHANES 2007–2012 ^1^.

Lifestyle Variable		TC	TG	HDL-C	LDL-C	Apo B
		mg/dL	mg/dL	mg/dL	mg/dL	mg/dL
Supplement use ^2^	Yes	203.2 ± 1.8 ^3,^**	117.7 ± 2.7	58.1 ± 0.9 **	121.5 ± 1.6	93.7 ± 1.1
	No	197.0 ± 1.1	123.4 ± 1.7	52.2 ± 0.5	120.1 ± 1.0	93.1 ± 0.8
	*r*^2^, *%* ^4^	<1	<1	3.09	<1	<1
Smoking ^5^	Low	203.1 ± 2.3	110.9 ± 3.2 **	59.2 ± 0.8 **	121.7 ± 2.2	92.3 ± 1.4
	Medium	197.8 ± 1.3	122.9 ± 2.6	53.6 ± 0.7	119.6 ± 1.1	92.9 ± 0.9
	High	198.5 ± 2.0	126.8 ± 2.5	51.7 ± 1.0	121.5 ± 1.8	94.7 ± 1.3
	*r*^2^, *%*	<1	<1	2.84	<1	<1
Alcohol consumption ^6^	None	202.9 ± 2.1 *	128.7 ± 3.3 *	51.7 ± 1.0 *	125.5 ± 1.6 **	97.0 ± 1.2 *
	>0 to <1 drink/day	197.2 ± 1.3	119.1 ± 1.7	54.0 ± 0.6	119.4 ± 1.1	92.1 ± 0.8
	1 to <2 drinks/day	202.8 ± 3.0	119.5 ± 5.8	57.1 ± 1.6	121.8 ± 2.2	93.9 ± 1.8
	≥2 drinks/day	202.8 ± 3.7	128.5 ± 6.4	57.5 ± 1.8	119.6 ± 3.8	94.9 ± 2.6
	*r*^2^, *%*	<1	<1	1.08	<1	<1
BMI ^7^	Underweight	189.4 ± 5.2 **	95.2 ± 7.5 **	62.6 ± 3.1 **	107.8 ± 4.7 **	82.5 ± 2.7 **
	Normal weight	193.9 ± 2.0	99.2 ± 1.7	61.3 ± 1.0	112.8 ± 1.5	85.4 ± 1.0
	Overweight	203.1 ± 1.5	128.0 ± 2.9	52.8 ± 0.8	124.8 ± 1.4	96.3 ± 1.0
	Obese	200.9 ± 1.9	139.4 ± 3.1	47.8 ± 0.6	125.2 ± 1.6	99.1 ± 1.1
	*r*^2^, *%*	1.15	7.22	11.92	3.09	6.40
Physical activity ^8^	No activity	200.5 ± 1.2	127.1 ± 2.0 **	52.7 ± 0.6 **	122.4 ± 1.1	95.4 ± 0.7 **
	0 to <500 MET-min/week.	199.9 ± 3.5	121.7 ± 3.2	54.0 ± 1.0	121.7 ± 2.7	93.3 ± 1.6
	500 to <1000 MET-min/week.	199.2 ± 3.6	117.2 ± 5.3	56.7 ± 1.1	119.0 ± 2.6	92.0 ± 2.0
	≥1000 MET-min/week.	196.5 ± 1.9	114.4 ± 2.6	55.7 ± 1.1	117.9 ± 2.0	90.6 ± 1.2
	*r*^2^, *%*	<1	<1	<1	<1	<1

^1^ Data are from the National Health and Nutrition Examination Surveys. All data except for sample size are weighted accounting for the complex study design according to the directions of the National Center for Health Statistics. Biomarker levels represent mean ± SEM. The total *n* size was 2495. TC, total cholesterol; TG, triglyceride; HDL-C, HDL cholesterol; LDL-C, LDL cholesterol; Apo B, apolipoprotein B; ^2^ reported taking supplement containing iodine within the past 30 days; ^3^ *p* values were based on Wald F test, which tests whether at least 1 of the means across the lifestyle variable categories is significantly different (* *p* < 0.05, ** *p* < 0.01); ^4^ values for *r*^2^ are based on model 1, simple linear regression, by categories as shown; ^5^ smoking status defined by a serum cotinine concentration (low: <0.015 mg/L; medium: 0.015 to <10 mg/L; high: ≥10 mg/L); ^6^ calculated as average daily number of drinks/day [(frequency × quantity)/365.25]; 1 drink ≈ 15 g ethanol; ^7^ underweight: <18.5 kg/m^2^; normal weight: 18.5 to >25 kg/m^2^; overweight: 25 to <30 kg/m^2^; and obese: ≥30 kg/m^2^; ^8^ calculated as total MET (metabolic equivalent task minutes)-min/week from self-reported leisure-time physical activities.

**Table 3:** Serum lipid profiles by urinary iodine concentration in US women, NHANES 2007–2012 ^1^.

Serum Lipids	Women	Low UIC, <10th Percentile (*n* = 148)	UIC ≥10th Percentile (*n* = 1008)	*p* Value
	Age (years)	mean ± SEM ^2^	mean ± SEM	
TC	20–39	188.6 ± 6.1	193.2 ± 4.6	0.2699
	40–59	205.6 ± 6.6	207.7 ± 4.5	0.7754
	≥60	231.6 ± 7.3	215.4 ± 4.3	0.0052 **
TG	20–39	101.1 ± 13.9	90.7 ± 8.1	0.2493
	40–59	110.3 ± 11.2	118.0 ± 10.2	0.3686
	≥60	82.39 ± 18.3	99.9 ± 10.3	0.2041
HDL-C	20–39	62.5 ± 2.5	62.5 ± 2.2	0.9948
	40–59	65.1 ± 2.8	67.7 ± 1.9	0.2380
	≥60	67.3 ± 3.8	67.4 ± 2.8	0.9801
LDL-C	20–39	106.2 ± 5.0	102.0 ± 4.3	0.3677
	40–59	118.9 ± 5.4	117.9 ± 4.0	0.8635
	≥60	151.8 ± 5.6	132.2 ± 3.2	0.0011 **
Apo B	20–39	81.9 ± 5.1	78.9 ± 4.1	0.3305
	40–59	92.4 ± 4.9	95.1 ± 3.8	0.4881
	≥60	107.8 ± 5.6	96.2 ± 2.9	0.0103 *

^1^ Data are from the National Health and Nutrition Examination Surveys. All data except for sample size are weighted accounting for the complex study design according to the directions of the National Center for Health Statistics. The total *n* size was 2495. UIC, urinary iodine concentration; TC, total cholesterol; TG, triglyceride; HDL-C, HDL cholesterol; LDL-C, LDL cholesterol; Apo B, apolipoprotein B; ^2^ weighted mean ± SEM. * *p* < 0.05, ** *p* < 0.01.

**Table 4:** Prevalence of dyslipidemia of adults in relation to urinary iodine concentration in US adults, NHANES 2007–2012 ^1^.

Dyslipidemia	Model	Low UIC, <10th Percentile (*n* = 249)	UIC ≥10th Percentile (*n* = 2246)	*p* Value ^2^
		AOR (95% CI)	Referent	
Elevated TC (>200 mg/dL)	1	1.35 (0.96–1.89)	1.00	0.0834
	2	1.42 (0.98–2.08)	1.00	0.0666
Elevated TG (>150 mg/dL)	1	0.76 (0.51–1.13)	1.00	0.1652
	2	0.93 (0.63–1.37)	1.00	0.7098
Lowered HDL-C (<40 mg/dL in men, <50 mg/dL in women)	1	0.64 (0.47–0.86)	1.00	0.0040 **
	2	0.92 (0.65–1.30)	1.00	0.6329
Elevated LDL-C (>130 mg/dL)	1	1.41 (1.04–1.92)	1.00	0.0283 *
	2	1.63 (1.16–2.28)	1.00	0.0054 **
Lowered HDL/LDL ratio (<0.4)	1	0.88 (0.65–1.19)	1.00	0.3991
	2	1.71 (1.09–2.67)	1.00	0.0199 *
Elevated Apo B (>130 mg/dL)	1	0.98 (0.50–1.91)	1.00	0.9567
	2	0.98 (0.46–2.08)	1.00	0.9505

^1^ Data are from the National Health and Nutrition Examination Surveys. All data except for sample size are weighted accounting for the complex study design according to the directions of the National Center for Health Statistics. Multiple logistic regression analysis was performed to estimate odds ratio for dyslipidemia for subjects from the NHANES 2007–2012 in two models: unadjusted (model 1) and adjusted for sex, age, race/ethnicity, education, income, supplement use, smoking, alcohol consumption, BMI, physical activity (model 2). UIC, urinary iodine concentration; AOR, adjusted odds ratio; 95% CI, 95% confidence interval. TC, total cholesterol; TG, triglyceride; HDL-C, HDL cholesterol; LDL-C, LDL cholesterol; Apo B, apolipoprotein B; ^2^ *p* value obtained from multiple logistic regression model with diagnosis of dyslipidemia as the outcome variables (* *p* < 0.05, ** *p* < 0.01).

## Supplementary Materials

The following are available online at http://www.mdpi.com/2072-6643/8/3/171/s1, Table S1: Sociodemographic and lifestyle characteristics of study subjects, NHANES 2007–2012, overall and by urinary iodine concentration, Table S2, Serum lipid profiles by urinary iodine concentration in US men, NHANES 2007–2012.
