# Supplementary material for: Low Urinary Iodine Concentrations Associated with Dyslipidemia in US Adults
Source: Nutrients. 2016 Mar 17;8(3):171. doi: 10.3390/nu8030171 (PMC4808899; doi:10.3390/nu8030171)
Supplement: Supplementary file 1 [file nutrients-08-00171-s001.docx]

**Supplementary Materials: Low Urinary Iodine Concentrations Associated with Dyslipidemia in US Adults**

Kyung Won Lee, Dayeon Shin and Won O. Song

**Table S1.** Sociodemographic and lifestyle characteristics of study subjects, NHANES 2007–2012 ^1^, overall and by urinary iodine concentration.

|  |  | **Total** | | **Low UIC,  <10th Percentile** | | **UIC  ≥10th Percentile** | | **Chi-Square  *p* Value ^2^** |
| --- | --- | --- | --- | --- | --- | --- | --- | --- |
|  |  | ***n*** | ***Wt'd %*** | ***n*** | ***Wt'd %*** | ***n*** | ***Wt'd %*** |  |
| Total | | 2495 | 100.0 | 249 | 10.0 | 2246 | 90.02 |  |
| Median UIC (µg/L) | | 144.4 (129.3–159.5) | | 31.9 (29.4–34.5) | | 150.7 (142.0–159.4) | |  |
| *Demographic characteristics* | |  |  |  |  |  |  |  |
| Sex | Men | 1339 | 50.9 | 101 | 35.8 | 1238 | 52.8 | 0.0004 ** |
|  | Women | 1156 | 49.1 | 148 | 64.2 | 1008 | 47.2 |  |
| Age | 20–39 years | 993 | 41.7 | 114 | 43.7 | 879 | 41.4 | 0.3390 |
|  | 40–59 years | 907 | 42.7 | 84 | 44.3 | 823 | 42.5 |  |
|  | ≥60 years | 595 | 15.6 | 51 | 12.0 | 544 | 16.0 |  |
| Race/ethnicity | NHW | 1155 | 72.2 | 122 | 74.7 | 1033 | 69.9 | 0.0519 |
|  | NHB | 491 | 8.7 | 47 | 8.8 | 444 | 10.5 |  |
|  | MA | 720 | 14.5 | 59 | 10.6 | 661 | 14.9 |  |
|  | Other | 129 | 4.6 | 21 | 5.9 | 108 | 4.7 |  |
| Education | Less than high school | 667 | 17.0 | 53 | 11.9 | 614 | 17.6 | 0.0079 ** |
|  | High school | 586 | 22.9 | 55 | 20.6 | 531 | 23.2 |  |
|  | More than high school | 1242 | 60.1 | 141 | 67.5 | 1101 | 59.2 |  |
| PIR | Low | 1198 | 33.8 | 114 | 30.3 | 1084 | 34.2 | 0.0858 |
|  | Medium | 558 | 22.9 | 49 | 18.6 | 509 | 23.5 |  |
|  | High | 739 | 43.3 | 86 | 51.1 | 653 | 42.4 |  |
| *Lifestyle characteristics* | |  |  |  |  |  |  |  |
| Iodine-containing upplement use ^3^ | Yes | 775 | 34.9 | 65 | 27.0 | 710 | 35.9 | 0.0208 * |
|  | No | 1720 | 65.1 | 184 | 73.0 | 1536 | 64.2 |  |
| Smoking ^4^ | Low | 491 | 21.5 | 50 | 23.2 | 441 | 21.3 | 0.4097 |
|  | Medium | 1253 | 49.9 | 118 | 44.8 | 1135 | 50.5 |  |
|  | High | 751 | 28.6 | 81 | 32.0 | 670 | 28.2 |  |
| Alcohol consumption ^5^ | None | 482 | 15.7 | 39 | 11.3 | 443 | 16.3 | 0.1161 |
|  | >0 to <1 drink/day | 1592 | 65.3 | 165 | 63.9 | 1427 | 65.4 |  |
|  | 1 to <2 drinks/day | 232 | 11.0 | 24 | 14.7 | 208 | 10.6 |  |
|  | ≥2 drinks/day | 189 | 7.9 | 21 | 10.0 | 168 | 7.7 |  |
| BMI ^6^ | Underweight | 67 | 2.4 | 9 | 2.4 | 58 | 2.5 | 0.0018 ** |
|  | Normal weight | 755 | 32.2 | 99 | 42.1 | 656 | 31.0 |  |
|  | Overweight | 852 | 34.7 | 85 | 37.6 | 767 | 34.4 |  |
|  | Obese | 821 | 30.7 | 56 | 17.9 | 765 | 32.3 |  |
| Physical activity ^7^ | No activity | 1286 | 43.6 | 114 | 36.1 | 1172 | 44.5 | 0.2158 |
|  | 0 to <500 MET-min/week | 339 | 15.8 | 37 | 15.5 | 302 | 15.8 |  |
|  | 500 to <1000 MET-min/week | 277 | 12.8 | 36 | 17.9 | 241 | 12.2 |  |
|  | ≥1000 MET-min/week | 593 | 27.8 | 62 | 30.4 | 531 | 27.5 |  |

^1^ Data are from the National Health and Nutrition Examination Surveys. All data except for sample size are weighted accounting for the complex study design according to the directions of the National Center for Health Statistics. Data values are reported as *n* (weighted percentage). Total of percentages may exceed 100 due to rounding. UIC, urinary iodine concentration; NHW, non-Hispanic white; NHB, non-Hispanic black; PIR, family poverty-income ratio (low: 0–1.85; medium: 1.85 < to 3.5; high: >3.5); ^2^ *p* value obtained from the Wald chi-square test (* *p* < 0.05, ** *p* < 0.01); ^3^ reported taking supplement containing iodine within the past 30 days; ^4^ smoking status defined by a serum cotinine concentration (low: <0.015 mg/L; medium: 0.015 to <10 mg/L; high: ≥10 mg/L); ^5^ calculated as average daily number of drinks/day [(frequency × quantity)/365.25]; 1 drink ≈ 15 g ethanol; ^6^ Underweight: <18.5 kg/m^2^; normal weight: 18.5 to >25 kg/m^2^; overweight: 25 to <30 kg/m^2^; and obese: ≥20 kg/m^2^; ^7^ Calculated as total MET (metabolic equivalent task minutes)-min/week from self-reported leisure-time physical activities.

**Table S2.** Serum lipid profiles by urinary iodine concentration in US men, NHANES 2007–2012 ^1^.

| **Serum Lipids** | **Men** | **Low UIC,  <10th Percentile  (*n* = 101)** | **UIC  ≥10th Percentile  (*n* = 1238)** | ***p* Value** |
| --- | --- | --- | --- | --- |
|  | **Age (Years)** | **Mean ± SEM ^2^** | **Mean ± SEM** |  |
| TC | 20–39 | 183.2 ± 5.6 | 81.3 ± 3.2 | 0.7500 |
|  | 40–59 | 218.6 ± 11.1 | 199.1 ± 4.6 | 0.0826 |
|  | ≥60 | 206.6 ± 11.1 | 210.1 ± 6.5 | 0.7476 |
| TG | 20–39 | 120.5 ± 15.6 | 120.7 ± 9.9 | 0.9831 |
|  | 40–59 | 119.3 ± 18.6 | 130.8 ± 15.1 | 0.4144 |
|  | ≥60 | 126.7 ± 18.4 | 116.3 ± 7.2 | 0.5247 |
| HDL-C | 20–39 | 47.3 ± 2.4 | 48.8 ± 1.6 | 0.5556 |
|  | 40–59 | 60.8 ± 4.0 | 53.9 ± 1.8 | 0.0548 |
|  | ≥60 | 53.4 ± 4.8 | 58.2 ± 3.0 | 0.3092 |
| LDL-C | 20–39 | 112.6 ± 4.3 | 109.1 ± 3.2 | 0.3891 |
|  | 40–59 | 134.6 ± 9.5 | 120.3 ± 2.6 | 0.1295 |
|  | ≥60 | 117.8 ± 10.7 | 123.6 ± 6.1 | 0.5887 |
| Apo B | 20–39 | 87.7 ± 4.0 | 86.2 ± 2.2 | 0.6619 |
|  | 40–59 | 101.9 ± 7.1 | 96.4 ± 2.8 | 0.4205 |
|  | ≥60 | 96.9 ± 7.0 | 99.2 ± 2.9 | 0.7387 |

^1^ Data are from the National Health and Nutrition Examination Surveys. All data except for sample size are weighted accounting for the complex study design according to the directions of the National Center for Health Statistics. The total *n* size was 2495. UIC, urinary iodine concentration; TC, total cholesterol; TG, triglyceride; HDL-C, HDL cholesterol; LDL-C, LDL cholesterol; Apo B, apolipoprotein B; ^2^ Weighted mean ± SEM.
